# Analyte Importance Analysis in Machine Learning-Based Detection of Wrong-Blood-in-Tube Errors Using Complete Blood Count Data

**DOI:** 10.3390/jpm15090404

**Published:** 2025-09-01

**Authors:** Barış Gün Sürmeli, René Staritzbichler, Clemens Ringel, Saleem Al-Dakkak, Helene Dörksen, Thorsten Kaiser

**Affiliations:** 1 Technische Hochschule Ostwestfalen-Lippe, Institute Industrial IT, 32657 Lemgo, Germany; helene.doerksen@th-owl.de; 2 University Institute for Laboratory Medicine, Microbiology and Clinical Pathobiochemistry, University Hospital OWL, Bielefeld University, Campus Klinikum Lippe, 32756 Detmold, Germany; rene.staritzbichler@klinikum-lippe.de (R.S.); saleem.aldakkak@klinikum-lippe.de (S.A.-D.); thorsten.kaiser@uni-bielefeld.de (T.K.)

**Keywords:** wrong blood in tube (WBIT), laboratory medicine, machine learning, feature importance, complete blood count (CBC), pre-analytical error detection, clinical decision support, explainability

## Abstract

**Background**: Wrong blood in tube (WBIT) is a critical pre-analytical error in laboratory medicine in which a blood sample is mislabeled with the wrong patient identity. These errors are often undetected due to the limitations of current detection strategies (e.g., delta checks). **Methods**: We evaluated Random Forest models for WBIT detection and conducted a detailed analyte importance analysis. In total, 799,721 samples from a German tertiary care center were analyzed and filtered for applicability. Model input features were derived by pairing consecutive same-patient samples for non-WBIT cases, simulating WBIT by pairing samples from different patients, and computing per-analyte first-order differences for each pair. We exhaustively searched all subsets of nine CBC analytes and evaluated models using F1 score, AUC, sensitivity, and PPV. Analyte importance was assessed via SHAP, permutation, and impurity decrease. **Results**: Models using as few as three analytes (MCV, RDW, MCH) reached F1 scores above 90%, with performance plateauing beyond six analytes. MCV and RDW were consistently top-ranked. Two-dimensional and three-dimensional visualizations revealed interpretable decision boundaries. **Conclusions**: Findings demonstrate that robust WBIT detection is achievable using a minimal subset of CBC analytes, offering a practical, interpretable, and broadly generalizable ML-based solution suitable for diverse clinical environments.

## 1. Introduction

Laboratory diagnostics plays a pivotal role in nearly all medical treatments, providing critical data that informs clinical decisions and patient management [1]. However, the reliability of these diagnostics can be compromised by pre-analytical errors—among the most serious of which is the wrong blood in tube (WBIT) error, where the blood in a sample tube does not originate from the patient whose name is printed on the label. This type of error can have serious clinical consequences, including misdiagnosis and inappropriate treatment. WBIT errors are estimated to occur in approximately 1 out of every 1000 samples, although this figure is likely an underestimation due to substantial under-reporting [2,3,4].

Several strategies have been implemented in clinical practice to address the issue of WBIT errors. Pre-analytical approaches, such as staff training and manual cross-checks, are designed to prevent such errors from occurring. In contrast, post-analytical measures, including automated delta checks and both technical and medical validation, aim to detect WBIT cases before they result in clinical harm.

However, the effectiveness of these methods in reducing or detecting WBIT errors has been shown to be limited [2]. The identification is challenging because of the high number of samples to be validated by laboratory personnel. Therefore, such errors often remain undetected or are identified only after a delay—potentially leading to serious patient harm [5].

Contemporary electronic solutions for the prevention and detection of WBIT, such as the implementation of two-dimensional barcode identification systems [6] and RFID scanning technologies [7], have proven effective. However, their widespread adoption is limited by the substantial costs and time requirement associated with implementation and ongoing maintenance [2].

Recently, data-driven artificial intelligence (AI) approaches, particularly machine learning (ML) models, have been increasingly applied across various medical domains [8,9,10]. In laboratory medicine, where large volumes of data are generated and managed through Laboratory Information Systems (LISs), ML models offer the potential to identify patterns in patients’ blood test results [11]. These patterns can help alert medical professionals when new results deviate significantly from what would be expected, as shown in studies such as [12,13]. For example, when two samples from the same patient show unusually large or inconsistent differences, it may indicate a pre-analytical error such as a WBIT event.

ML-based WBIT detection systems have been developed in several recent studies and have demonstrated superior performance compared to single-analyte delta checks [14] and manual result review [15,16]. Although ML-based techniques developed to date have achieved impressive performance in detecting WBIT errors, a comprehensive feature analysis that investigates the impact of individual analytes on model performance has not yet been conducted. Such analysis is crucial because the availability of measured parameters in patient samples can vary significantly both within and across clinical institutions. Without this level of insight, the interpretability of the trained models remains limited, and the mechanisms enabling their accurate predictions are not well understood. This lack of transparency reflects a broader challenge associated with applying ML methods in medical contexts, where explainability is often as important as accuracy.

In this study, a comprehensive feature analysis was conducted to evaluate ML-based WBIT detection using classifier models trained on a dataset obtained from a German tertiary care center. The models were trained exclusively on complete blood count (CBC) data, as these are widely used in clinical practice. Exhaustive search was performed to identify the most effective analyte combinations for different subgroup sizes. In this approach, analyte combinations were systematically ranked based on the classification performance of the models trained with them.

## 2. Materials and Methods

### 2.1. Python and Libraries

All scripts were implemented with Python 3.9. The library scikit-learn 1.2 [17] was used for the training and evaluation of the machine learning models, matplotlib 3.6.3 for the visualization, pandas 1.5.2 for the data manipulation, and mlxtend 0.23.1 for the exhaustive feature selection.

### 2.2. Dataset

The clinical dataset used in this study was collected from a German tertiary care center, Klinikum Lippe Detmold (KLD), and includes 799,721 anonymized blood samples from 154,998 patients over a period of 1585 days. This study was approved by the Ethics Commission of the University of Münster under the reference number 2024-064-f-S. Different combinations of analytes were determined in the laboratory for the samples, according to the parameters that were ordered in the clinic. For this study, the analysis was limited to the analytes included in the complete blood count (CBC) panel (without differential blood count), as this is the most common panel in clinical practice.

The CBC was performed using a flow cytometry-based hematology analyzer (Advia 2120i, Siemens Healthineers, Erlangen, Germany), which reported the following nine analytes: red blood cells (ERY), hemoglobin (HB), hematocrit (HK), mean corpuscular hemoglobin (MCH), mean corpuscular volume (MCV), mean corpuscular hemoglobin concentration (MCHC), red blood cell distribution width (RDW), platelets (PLT), and white blood cells (LEUKO). Of these, ERY, LEUKO, PLT, MCV, RDW, and HB were measured directly. HK, MCH, and MCHC were calculated by the analyzer based on the following formulas: HK=ERY×MCV, MCH=HB/ERY, MCHC=HB/HK.

The baseline characteristics of the dataset after filtering are summarized in Table 1, including means, standard deviations, and value ranges for all analytes. An overview of sample distribution by patient sex, sampling frequency, and inter-sample intervals is provided in Table 2.

### 2.3. Filtering

**Figure 1:**
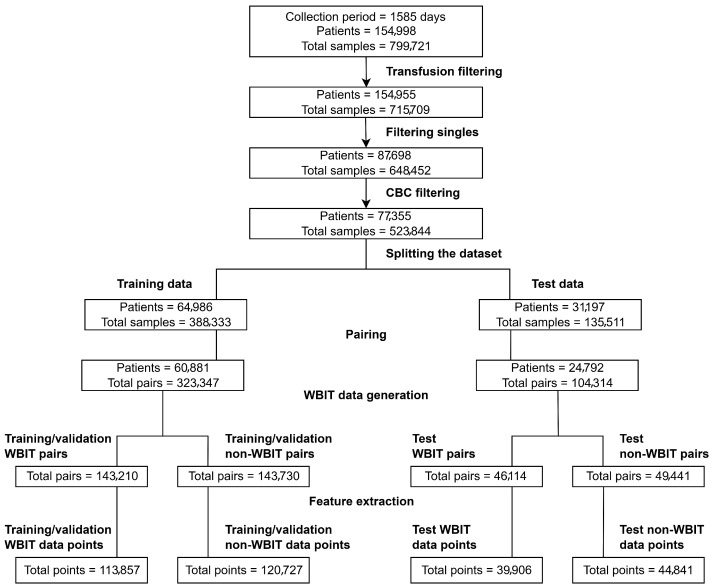
Overview of the data preprocessing workflow.

The dataset was filtered in the following steps: **Transfusion filtering**: Samples from patients who had received a blood transfusion were excluded if they were collected within 90 days following the transfusion. **Filtering singles**: Patients with only one sample in the dataset were removed. **Complete blood count (CBC) filtering**: Samples that did not contain all nine CBC analytes measured by the laboratory at KLD were excluded. These steps, along with the corresponding sample and patient counts after each filtering stage, are visualized in Figure 1.

### 2.4. Splitting the Dataset

The dataset was split such that samples from the first 1269 days were assigned to the training/validation set, and the remaining 316 days (approximately 20% of the total duration) were assigned to the test set. While the train–test split respected temporal order to prevent information leakage, patient-level separation was not enforced, and some patients may appear in both sets. Although this could introduce a mild overestimation of performance, it also reflects a realistic deployment setting where models are periodically retrained and encounter both new and previously seen patients. Future work may additionally assess performance under patient-disjoint conditions to quantify this effect more precisely. All preprocessing steps following the split were performed independently for the training/validation and test sets.

### 2.5. Pairing

**Figure A1:**
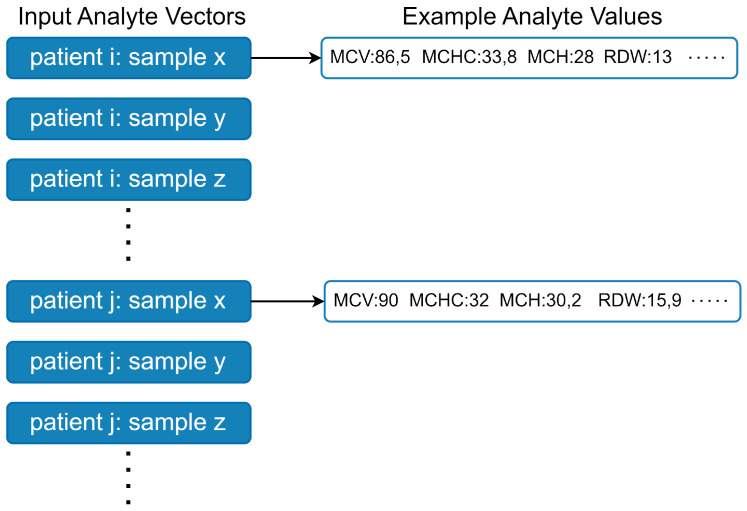
Data is input to the model as analyte vectors. Each analyte vector corresponds to a sample, while there are multiple samples from multiple patients. The example values are randomly generated for visualization purposes and they do not belong to patients.

**Figure A2:**
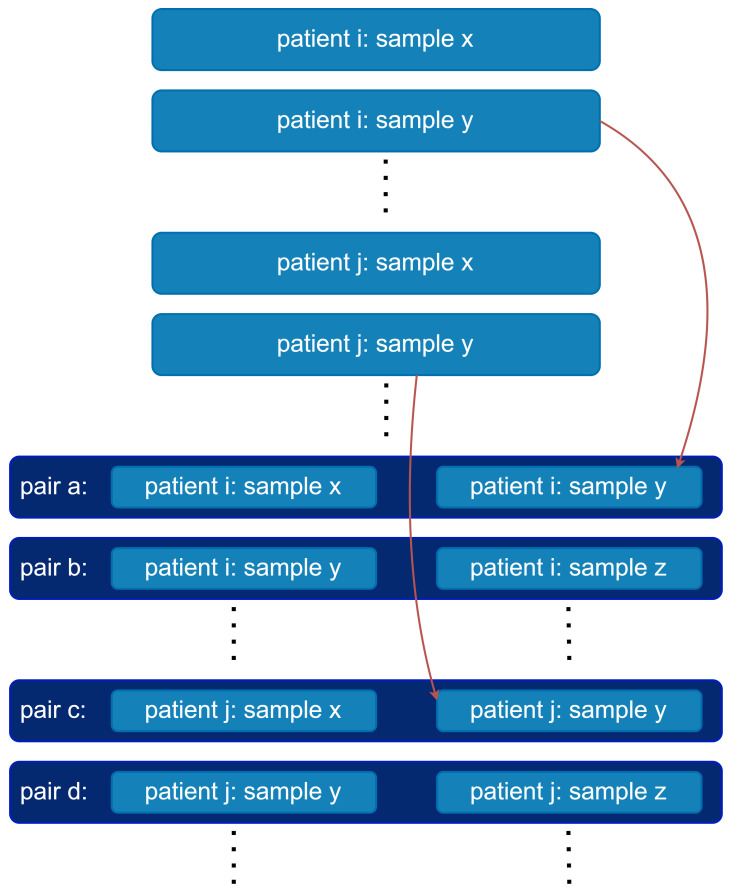
Pairing of analyte vectors. For each patient, their consecutive analyte vectors are concatenated to form pairs.

The dataset contains multiple analytes measured for each blood sample, which together form analyte vectors (see Figure A1). A common approach in ML-based WBIT detection is to train binary classifiers, where a given analyte vector is classified into one of two categories: WBIT or non-WBIT. While class labels for training could theoretically be generated by swapping portions of analyte vectors from different patients and labeling them as WBIT, such synthetic labels do not reflect meaningful patterns that the model can learn, as there is no inherent relationship between the features and the assigned classes. To address this, it is standard practice in the literature to use analyte vector pairs (see Figure A2) as input. A pair is labeled as WBIT if it consists of vectors from samples belonging to two different patients, and as non-WBIT if both samples originate from the same patient. This setup enables the model to learn distinguishing patterns between pairs that represent the same individual and those that do not.

### 2.6. Data Quality

It is likely that there are some naturally occurring WBIT errors in our dataset that were not recognized in the clinical setting. A future study is planned to address the identification and removal of such cases using outlier elimination techniques, such as those described in [18]. We also intend to evaluate an iterative approach in which a trained model is used to filter out potential WBIT cases, followed by retraining the model on the refined dataset. For the purposes of this study, we assumed that the training data did not contain any undetected WBIT errors.

### 2.7. Non-WBIT Pair Generation

In order to generate non-WBIT pairs, we did not form random pairs. Instead, in this study, we paired consecutively drawn samples from each patient (see Figure A2). This approach offers two key advantages: (1) it minimizes variation within non-WBIT pairs by reducing time-dependent biological drift between samples; and (2) it supports practical deployment, as it enables straightforward construction of prediction inputs by pairing each new sample with the most recent previous sample from the same patient. Consecutive samples separated by more than 30 days were excluded from pairing.

### 2.8. WBIT Pair Generation

**Figure A3:**
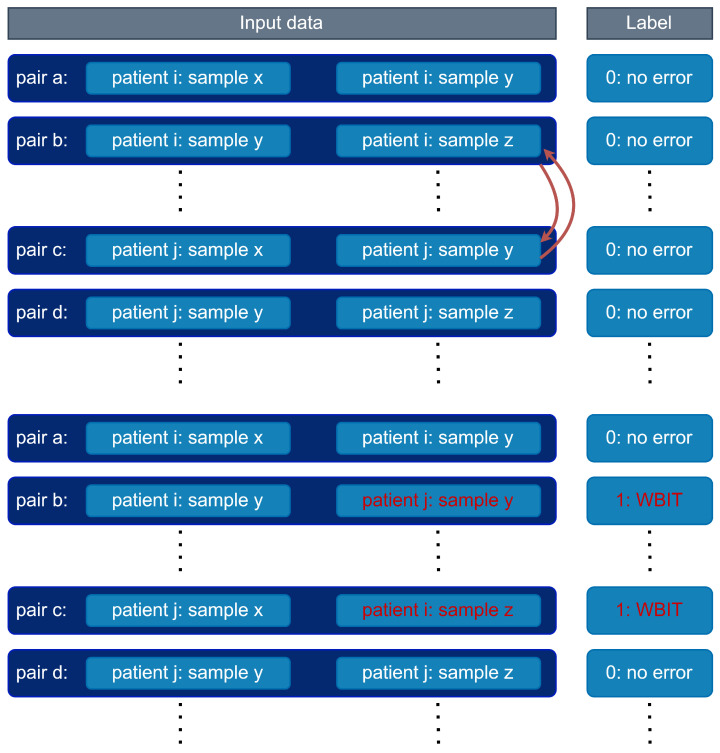
WBIT generation. Second analyte vectors in the pairs of different patients are swapped. Pairs are picked randomly among the pairs where the corresponding samples of the second analyte vectors in the pairs are drawn in the same day and ward.

We generated/simulated WBIT data using the following steps:

- Temporal segmentation: The dataset was divided into 24-h intervals based on sample collection timestamps.
- Ward-based grouping: Within each 24-h interval, analyte vector pairs were grouped based on the ward associated with the second vector (i.e., the more recent sample in the pair).
- Pair swapping to simulate WBIT: For each (day × ward) group, we randomly selected pairs of analyte vector pairs, each from different patients. In each selected pair, the second analyte vectors were swapped to simulate a WBIT error (see Figure A3).
- This process was repeated until approximately 50% of the pairs in each (day × ward) group were converted to WBIT-labeled cases.
- Groups with fewer than two distinct patients were excluded from the simulation process. Additionally, slight class imbalance may occur as (day × ward) groups with an odd number of eligible pairs retain one unmatched non-WBIT pair, which is not discarded.

The data preparation steps including preprocessing and WBIT generation are visualized in Figure 1.

### 2.9. Feature Generation

In addition to the analyte vector pairs described in Section 2.2, input data points are often further processed or extended with additional features in the literature. Several approaches have been proposed: ref. [19] used consecutive analyte vectors formatted not as pairs but as quadruples, as illustrated in Figure A2; ref. [15] incorporated patient metadata such as age and sex; ref. [14] included absolute changes and velocity; ref. [20] combined absolute and relative changes; and ref. [21] further integrated the time difference between the timestamps of the two samples in each pair.

**Figure A4:**
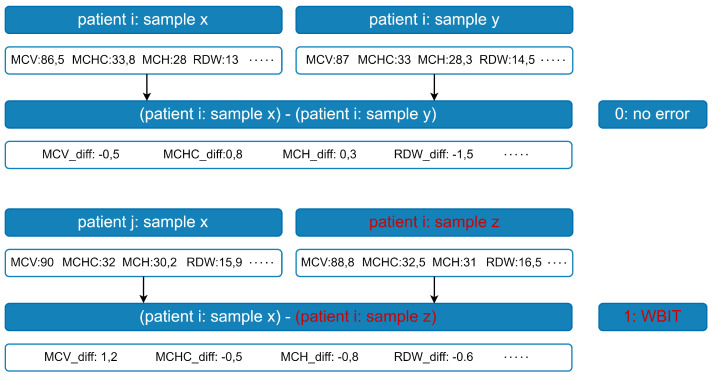
Feature generation. First order differences are calculated for each analyte vector pair; the first vector in each pair is element-wise subtracted from the second.

Since the focus of this study was exclusively on analyte values rather than extensive feature engineering, a simple and robust method was preferred. Specifically, first-order differences were computed between the values of the two analyte vectors in each pair: the values of the first vector were subtracted element-wise from those of the second to obtain the feature set (see Figure A4). This transformation was applied to all analyte vector pairs for each patient, resulting in 9-dimensional feature vectors per pair.

### 2.10. Model Training

Random Forest classifiers (RFCs), introduced in [22], are widely used across diverse domains and have shown strong performance in WBIT detection tasks [15,16]. In preliminary experiments, we also evaluated logistic regression, support vector machines (SVMs), and dense feedforward neural networks. These models were more sensitive to hyperparameter settings and, despite tuning efforts, yielded inferior performance and/or lower robustness compared to RFCs. Given their efficient training, relatively low memory usage, minimal hyperparameter complexity, and high interpretability, Random Forests were selected for this study. Throughout the remainder of this paper, the term model refers specifically to Random Forest classifiers.

All models were implemented using the scikit-learn library. Each Random Forest classifier was trained with class-balanced weights to account for label imbalance. All other hyperparameters were left at their default values.

## 3. Results

### 3.1. Analyte Importance Analysis

In order to explore the full range of predictive performance, an *exhaustive feature search* was conducted. For each *n* in the range 1≤n≤9, all possible combinations of *n* analytes were used to train models, and their performance was compared.

For each evaluated feature combination, 5-fold cross-validation was performed using the training/development set (see Figure 1), and average scores were computed across the folds. Model performance was assessed and ranked using the F1 score, a widely adopted metric in binary classification tasks that represents the harmonic mean of positive predictive value (PPV) and sensitivity.

While exhaustive search is effective for identifying high-performing analyte combinations, its application becomes impractical for larger analyte sets. In such cases, a more scalable approach is to perform an analyte importance analysis using dedicated metrics. These metrics offer complementary insights into the role of each analyte, including its relative impact on model performance, the magnitude of that impact, and the conditions under which the analyte proves most beneficial.

Accordingly, we applied feature importance analysis to the best-performing models identified through exhaustive search for each *n*. Analytes were ranked based on their contribution to the classification decisions across all samples. Three different importance metrics were used:

SHapley Additive exPlanations (SHAP) [23] is a method based on game theory that assigns each “player” (analyte) a fair “payout” proportional to its contribution to the final prediction. In the context of tree-based models such as RFCs, the Tree SHAP algorithm [24] is used to efficiently compute SHAP values by accumulating the marginal contribution of each feature along the path from the root to the leaf during classification.

Permutation Importance (PI) [25] estimates the influence of each feature by measuring the decrease in model performance when the values of that feature are randomly shuffled while keeping all others fixed. A greater drop in performance indicates higher importance of the shuffled feature.

Mean Decrease in Impurity (MDI) [22] is a tree-specific metric that quantifies feature importance based on how much a given feature contributes to reducing impurity in the decision tree. Impurity reflects how mixed the class labels are within a node; features that lead to greater impurity reduction during splits are considered more important. MDI is computed by summing the impurity reductions across all splits involving the feature and averaging the result.

Some analytes perform well individually, while others exhibit synergistic effects when used in combination. MDI and SHAP primarily capture the individual importance of features, whereas PI can better reflect the utility of features in interaction with others. For this reason, all three metrics were used to obtain a more comprehensive and reliable assessment of analyte importance.

Accuracy, positive predictive value (PPV), sensitivity, area under the receiver operating characteristic curve (ROC AUC), and F1 scores of the top five performing models for each analyte set size (*n*) are reported in Table 3.

**Figure 2:**
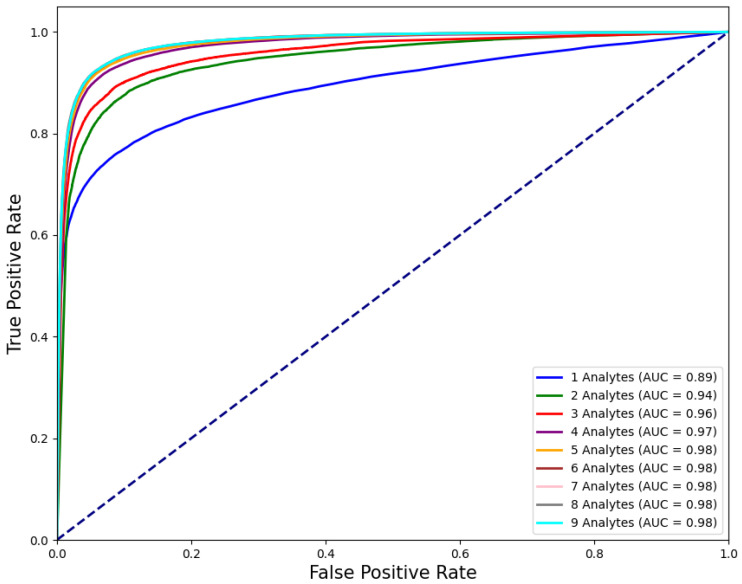
ROC curves of the models that are trained on analyte combinations that yield the best performance for each *n*.

The best performing models for each *n* were further evaluated on the test set (see Figure 1) as follows. Receiver operating characteristic (ROC) curves are shown in Figure 2.

**Figure 3:**
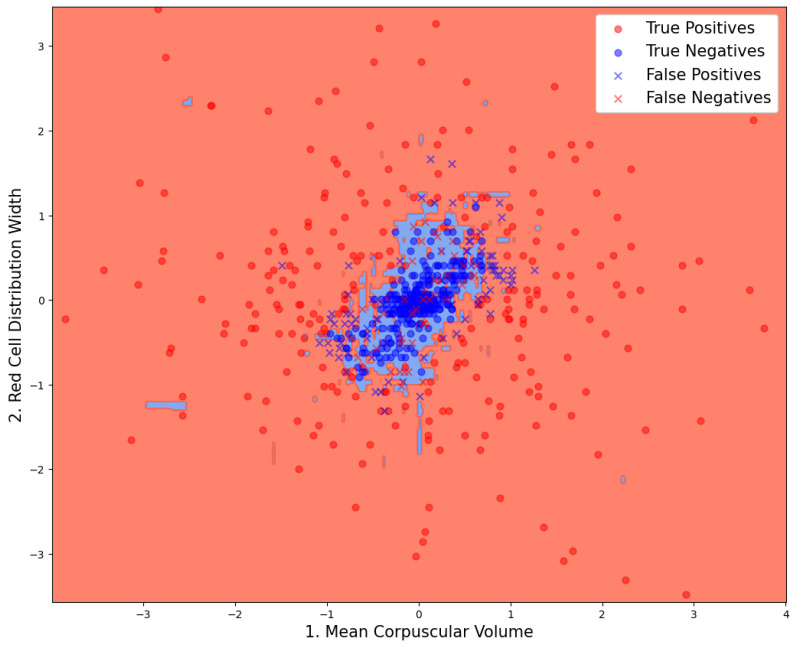
Data point distribution on the model that is trained on the combination of analytes that yield the best performance for n=2. Classification results and decision boundaries of the model can be seen.

**Figure 4:**
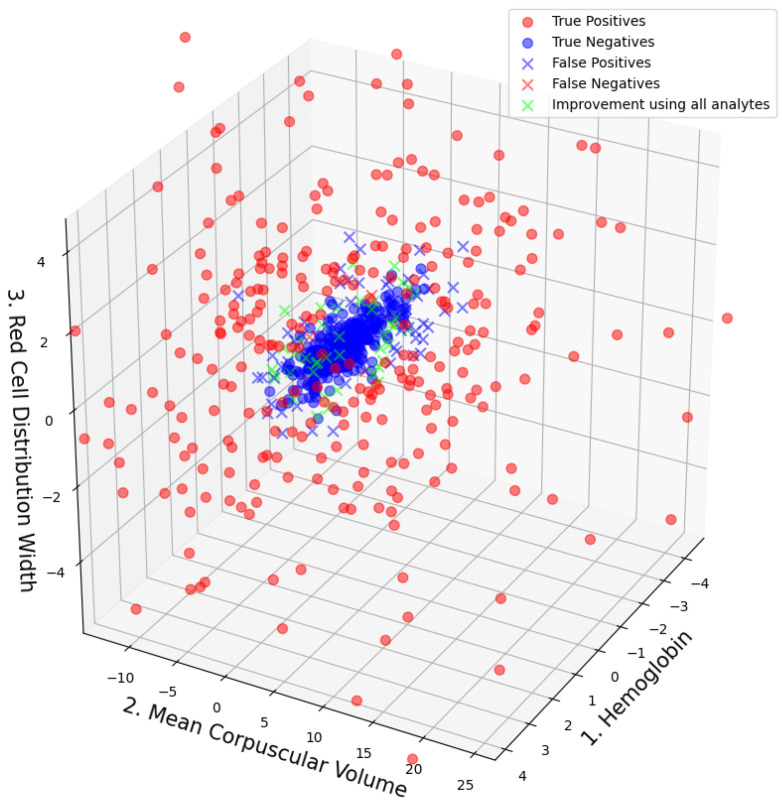
Data point distribution on the model that is trained on the combination of analytes that yield the best performance for n=3. Classification results and the data points that would have been correctly classified if the used model was trained on all available analytes are plotted in green.

F1 scores, ROC AUC values, and their bootstrap confidence intervals (bsCIs), along with PPV at sensitivities of 0.8 and 0.5 under an assumed WBIT error rate of 0.005 (reflecting a realistic WBIT rate in the test set), are summarized in Table 4. The corresponding metrics are reported using the abbreviations PPV@S0.8E0.005 and PPV@S0.5E0.005, respectively. Feature importance metrics are presented in Table 5 and for the best models trained using two and three analytes; the distribution of the data points and the associated class predictions are visualized in Figure 3 and Figure 4, respectively.

### 3.2. Analyte Importance Rankings

In Table 3, the analytes MCV and RDW consistently appear among the highest ranking combinations across different values of *n*. MCH is among the top-performing combinations only for n≥5, where it typically ranks second or third. Notably, MCV and RDW are the only analytes that appear in the top three across all importance metrics and remain part of the best-performing combinations for every value of *n*. In Table 5, which is sorted by MDI, a clear drop in both MDI and SHAP values is observed for analytes ranked below MCV, RDW, and MCH for n≥6. When sorting by PI, similar drops occur for analytes ranking below MCV and RDW.

### 3.3. Model Performance Using Smaller Analyte Sets

As shown in Figure 3, model performance improves with the number of analytes used, as expected. However, this improvement plateaus beyond n=6 (p≥0.05; pairwise statistical comparisons between the top-performing combinations of consecutive analyte set sizes were conducted using a paired *t*-test on the cross-validation fold scores; accuracy, F1, ROC AUC, PPV and sensitivity) for all evaluated metrics except sensitivity, which continues to show significant gains up to n=7, after which no substantial improvements are observed. Even with only n=3, the F1 score exceeds 90%, highlighting that strong classification accuracy can be achieved with a minimal set of features. This trend is consistent with the PI values reported in Table 5, where analytes ranked below the top five for n≥5 have zero importance. This suggests that these lower-ranking analytes contribute to performance only in combination with more influential features.

Performance variation between top-performing analyte combinations (see Table 5) within each fixed set size was statistically significant (p<0.05; pairwise comparisons between consecutive top-five combinations within each set size were conducted using a paired *t*-test on cross-validation F1 scores) for smaller sets (e.g., n=1 and n=2), while such differences diminished for n≥5. This suggests that performance is more sensitive to analyte selection when fewer features are used, whereas models with larger analyte sets tend to converge to similar performance levels across combinations.

In certain cases, models trained on a relatively smaller number of features can outperform those trained on the full feature set, as they may generalize better and are less prone to overfitting. The results in Table 4 reflect this phenomenon: PPV values peak at n=8, while ROC AUC and F1 scores reach their highest values at *n* = 7, with a slight decline observed for larger values of *n*. Additionally, sensitivity is consistently lower than PPV, particularly for n<5, indicating an imbalance in the error rates that favors precision over recall in models with fewer analytes.

### 3.4. Explanations by Visualizations

In Figure 3 and Figure 4, the true class labels of the data points are distinguished by color: samples belonging to the WBIT class are shown in red, while those from the non-WBIT class are shown in blue. In Figure 3, the decision regions learned by the random forest model for the two classes are visualized as light red and light blue areas. A new data point falling within the blue region would be classified as non-WBIT, and one in the red region as WBIT. Correctly classified data points are marked with circles (“o”), while misclassified points are indicated with crosses (“x”). In Figure 4, green crosses denote misclassified points that would have been correctly classified if the model had been trained on all available analytes (n=9).

## 4. Discussion

### 4.1. Analyte Importance

Recent reports highlight substantial variation in the composition of CBC reports across clinical sites, with the number and types of analytes differing significantly between institutions [26]. In this context, the ability to achieve strong model performance using small, well-chosen subsets of analytes—as demonstrated in Section 3.3—is particularly relevant. This feature reduction enhances generalizability across heterogeneous datasets and enables operational flexibility for deployment in constrained or embedded environments. Moreover, the resulting low-dimensional models improve interpretability, allowing for direct visualization of decision boundaries and model behavior. Finally, identifying compact, high-performing subsets offers scientific insights into which analytes contribute most strongly to WBIT detection—potentially informing future diagnostic heuristics.

The high predictive power of MCV, RDW, and MCH is consistent with findings from previous studies [27] and can be considered biologically plausible. This is because the models rely on differences between consecutive samples, and these analytes are known to have lower biological variation ratios within individuals, both compared to the within individual variation of other analytes and their own between individual variation [28]. These analytes also stand out with substantially higher feature importance scores compared to others, suggesting that they account for the majority of the predictive information.

As discussed in Section 2.2, HK, MCH, and MCHC are derived analytes and do not offer additional independent information. In theory, models trained exclusively on directly measured analytes should be sufficient for effective prediction. Nevertheless, including derived features can still be beneficial in practice—for instance, by simplifying model learning or acting as proxies for latent patterns that may not be directly captured by the original variables. This appears particularly relevant for MCH, which is calculated from HB and ERY.

It is important to note that the analyte rankings based on importance metrics do not always align with the combinations that yield the best predictive performance. For example, while MCH ranks among the most important features for n=9, it does not appear in the most predictive combinations for n<4 (see Figure 3). This discrepancy illustrates the limitation of relying solely on feature importance metrics when selecting subsets for model training.

Although exhaustive search is a powerful tool for identifying high-performing analyte combinations, it becomes computationally infeasible for larger values of *n* due to the combinatorial explosion. In order to maximize predictive performance under resource constraints, a more scalable strategy may be to first use importance metrics or feature selection algorithms like Boruta [29] on a model trained using all analytes to narrow the analyte pool to a smaller set with consistently high scores. Subsequently, exhaustive search can be applied within this reduced set to determine the optimal feature combination for a given *n*.

### 4.2. Model Behavior

Inspection of the misclassified data points in Figure 3 and Figure 4 reveals that most false positives are located near the cluster of true negatives. This is expected, as data points from both classes overlap in these regions, increasing the likelihood of classification errors. Although a direct visualization is not possible for n=9, it can be inferred from Figure 3 that the green data points—representing samples misclassified by the n=3 model but correctly classified by the n=9 model—are likely located farther from the cluster center in the higher-dimensional space. This spatial separation may allow the model trained on all nine analytes to distinguish such points more effectively. The boundaries between the blue and red decision areas in Figure 3 can be interpreted as complex, multi-analyte delta check rules. Each straight boundary segment corresponds to a single rule associated with the analyte to which it is perpendicular.

The models in this study produce class probabilities for each data point, representing the likelihood of the sample belonging to the WBIT class. A probability threshold (set to 0.5 throughout this study) is then used to assign class labels. This threshold directly influences the size of the decision regions: increasing the threshold reduces the size of the WBIT (blue) area, resulting in fewer false positives but more false negatives. The appropriate threshold should be chosen according to the laboratory’s tolerance for these trade-offs. False negatives, on the other hand, are primarily located within the central region of the true negative cluster in Figure 3. These errors appear more difficult to eliminate using only two analytes. It is plausible that, in these WBIT cases, the mismatched patients happened to have very similar blood profiles, making the error more difficult to detect. The consistently lower sensitivity compared to other scores observed in Figure 3 can be explained by the underlying data distribution (Figure 3 and Figure 4): the non-WBIT (negative) class forms a tightly clustered group, while the WBIT (positive) class is more dispersed. This asymmetry in spatial density increases the model’s tendency to misclassify WBIT data points, thereby lowering sensitivity.

### 4.3. Limitations and Outlook

#### 4.3.1. Applicability

In clinical practice, the set of analytes measured in each sample varies considerably depending on the laboratory tests ordered. While this study focused exclusively on complete blood count (CBC) data due to its clinical ubiquity and interpretability, prior work has employed a broader range of analyte sets. For example, ref. [19] incorporated both hematology and biochemistry markers, including liver enzymes and electrolytes; ref. [14] used metabolic parameters such as glucose, BUN, and creatinine; ref. [20] worked with standard panels like the basic and comprehensive metabolic panels; and ref. [16] relied on electrolyte–urea–creatinine (EUC) results. These studies demonstrate the feasibility of using diverse analyte sets for WBIT detection.

Our original dataset contained many samples in which only a subset of analytes was measured, reflecting the variability of test orders in routine clinical practice. Restricting the dataset to samples with complete CBC panels substantially reduces the overall dataset size, potentially discarding valuable information. ROC curves in Figure 2, and the ROC AUC and F1 scores in Table 4, show substantial performance gains up to n=6 (p<0.001; pairwise statistical significance between models of adjacent analyte set sizes was assessed using a paired *t*-test on per-iteration bootstrap estimates of F1 and ROC AUC scores). For larger *n* values, performance improvements become negligible or inconsistent across metrics, with no further statistically significant gains. This suggests that analytes that do not contribute to performance improvement could be excluded before filtering, thereby increasing the number of usable samples. Conversely, for analytes that do improve performance but are less frequently measured, imputation techniques such as those used in [30] could be employed to reconstruct missing values. This would enable the use of a dataset in the analyte vector format shown in Figure A1 before model training. Alternatively, models that can handle varying input dimensions could be used. For instance, the widely applied, tree-based XGBoost algorithm can adapt well via its sparsity aware splitting mechanism (see [31] Section 3.4). Another candidate is Graph Neural Networks [32], which can naturally model variable-sized and irregularly structured data by representing analytes and their relationships as nodes and edges in a graph. Future research should explore such adaptive modeling approaches to support WBIT detection across a broader range of clinical scenarios, where analyte availability may differ between samples.

The analyte importance analysis framework presented in this paper is not limited to CBC data. These techniques are also promising for identifying informative features in other laboratory panels and should be systematically evaluated in future work to assess their applicability beyond CBC analytes.

#### 4.3.2. Undetected WBIT in Training Data

This study assumed that the training data did not contain undetected WBIT errors. In reality, many WBIT cases may go unnoticed in clinical workflows. Ref. [3] estimated the rate of undetected WBIT errors at 3.17 per 1000 samples, compared to a detected error rate of 1.15, resulting in an estimated total error rate of 4.32 per 1000 samples. This poses a challenge for both training and evaluation, as undetected cases are incorrectly treated as normal, potentially distorting the model’s learning. As an unsupervised learning approach, outlier detection models such as Local Outlier Factor (LOF) [18] aim to identify anomalies in unlabeled data. Such models could be used as a preprocessing step to remove potentially mislabeled WBIT cases from the dataset, enabling the training of more robust ML models.

#### 4.3.3. Time Series

Similar to previous studies [14,15,16,20,21,30], only the most recent prior sample was used in this study to determine whether a current sample is a WBIT case. In contrast, ref. [19] employed analyte vector quadruples rather than pairs. Theoretically, all previously collected samples for a patient could be leveraged to extract richer temporal patterns in analyte value changes. However, due to computational limitations, this would require either machine learning algorithms capable of handling variable-length input—such as Long Short-Term Memory (LSTM) networks [33]—or the use of workaround strategies such as averaging all previous samples or combining separate models trained on inputs of different lengths.

The time threshold between consecutive samples used to form analyte vector pairs (see Section 2.5) varies across studies. For instance, ref. [30] used 6 days, ref. [20] used 10 days, and ref. [14] used 30 days, as adopted in this study, while ref. [19] did not apply a time threshold. To the best of our knowledge, only ref. [30] explicitly accounted for transfusion history by separately handling the data of patients who received blood transfusions within the past three months. In our study, such samples were removed entirely from the dataset (see Section 2.3). These parameters—time thresholds and transfusion exclusion criteria—are typically determined based on laboratory constraints or arbitrarily set. Future work should focus on systematically optimizing these parameters to maximize model performance while maintaining operational feasibility for clinical deployment.

#### 4.3.4. Higher Order Classification-Algorithmic Extensions

To date, most studies have framed WBIT detection as a binary classification problem. An idealized alternative would be a model capable of learning patient-specific analyte profiles and classifying samples directly as belonging to individual patients, rather than determining whether a sample is WBIT or not. While conceptually promising, this approach is impractical in real-world settings, as most patients in clinical databases have only one or two samples, which is insufficient for modeling individual longitudinal patterns. Nevertheless, certain long-term patients contribute a significantly higher number of samples (see Table 2). Future research could explore hybrid approaches that combine binary classification with patient-specific modeling, provided that enough patient-specific data are available.

As explained in Section 2.10, Random Forest classifiers were the only models trained in this study. Tree-based models such as Random Forests have been shown to perform particularly well on tabular datasets [34], such as the one used in this study (see Figure A1). More advanced tree-based algorithms like XGBoost have demonstrated outstanding performance in WBIT detection in prior studies [19,30]. Other widely used machine learning algorithms, including Neural Networks and Support Vector Machines (SVMs), have also been successfully applied in related work [14,15,20].

In addition to these traditional models, recent deep learning architectures specifically designed for tabular data—such as the FT-Transformer [35] and *SAINT* [36]—may offer further potential for WBIT detection due to their capacity to model complex interactions among features. However, the application of such deep learning models is computationally demanding. Their practical implementation in clinical settings depends heavily on the availability of sufficient computational resources within the laboratory environment.

#### 4.3.5. Clinical Considerations

The positive predictive value (PPV) scores achieved in this study (see Table 4) may not yet be sufficient for deployment in clinical laboratory settings. This is particularly important because a high number of false positives could undermine trust in the system, leading clinical staff to disregard valid alerts. Furthermore, ROC AUC values exceeding 99.99% have been reported in the literature [30], which is substantially higher than the maximum of 97.93% achieved in this study. Combining the analyte impact analysis presented here with state-of-the-art machine learning techniques could yield models with improved accuracy, reduced false alarm rates, and greater robustness across varying clinical environments.

Furthermore, all WBIT cases in this study were simulated, and therefore, PPV estimates are conditional on the assumed prevalence. We used a fixed prevalence of 0.5%, based on the estimate of 0.432% reported in [3] (see Section 4.3.2). Notably, ref. [19] also adopted a 0.5% assumed prevalence when estimating PPV. While this does not reflect confirmed WBIT events in our dataset, it provides a reasonable reference point for threshold calibration during deployment.

WBIT generation and simulation strategies also differ across studies. While many employ simple random swapping, more realistic methods—such as restricting swaps to samples collected from the same ward [19,21] or, as in this study, from the same ward and within a 24-h period—have been proposed to better reflect clinical conditions. Although realistic simulation is desirable, future research should investigate how different WBIT generation strategies affect classification performance.

An additional limitation is the exclusion of post-transfusion samples. While this step helped isolate analyte-driven variation and aligns with standard preprocessing in prior studies, it may limit generalizability in real-world settings where transfusions are frequent. Future work could explore whether including transfusion history—including timing and type—allows models to retain robustness in such contexts.

It is important to emphasize that, unlike in retrospective studies based on simulated WBIT errors, definitive confirmation of a WBIT event is not feasible in routine clinical practice. This limitation presents two major challenges: (1) accurately estimating the true incidence of WBIT errors, and (2) prospectively evaluating the performance of detection models. To address the latter, several strategies may be considered: (1) contacting the treating physician to assess whether drastic changes in analyte values between samples can be clinically explained—although this can be particularly difficult when the suspected WBIT case falls within or near the dense cluster of non-WBIT data points, (see Figure 3 and Figure 4); (2) examining additional analytes not used in model training, which may be inconclusive if the WBIT affects only specific sample tubes (e.g., an error in the EDTA tube used for CBC might not manifest in other tubes); (3) performing supplementary tests such as HbA1c, BSG, or blood group typing on the original sample, though this becomes impractical when the model is trained on analyte vector pairs (see Section 2.5), as the first sample is often no longer available; and (4) obtaining a follow-up sample to assess whether the model flags another WBIT alert—a strategy ultimately constrained by the model’s inherent limitations. Given these challenges, the uncertainty in identifying true WBIT cases must be acknowledged, and both WBIT incidence rates and model performance metrics should ideally be reported with appropriate upper and lower bounds to reflect this ambiguity.

A common strategy for estimating undetected WBIT rates involves focusing on cases flagged as suspicious and performing additional blood group testing. For example, in [3], WBIT rates were estimated based on the probability of two patients having the same ABO/Rh blood group, using blood group frequency data. In a prospective evaluation, ref. [30] selected the five samples with the highest predicted WBIT probability each week for follow-up blood group testing. Although this strategy is promising for both clinical integration and prospective assessment, further research is needed to reduce false alarm rates while also addressing the risk that valid alerts may be incorrectly dismissed due to the difficulty of confirming WBIT errors. Further details on possible methodologies for integrating ML-based WBIT detection into routine laboratory workflows can be found in [30]. Future work should aim to develop implementation frameworks that account for economic, organizational, and infrastructural constraints in diverse clinical environments.

#### 4.3.6. Explainability

While our analysis centers on global feature importance for model interpretability and analyte selection, future integration of local explanation techniques—such as per-sample SHAP value analysis—could further enhance clinical transparency by highlighting the specific analyte shifts driving individual predictions [37]. Such methods may assist laboratory staff in verifying WBIT alarms and support model refinement by providing insight into misclassifications.

As the field moves toward clinical deployment of interpretable ML systems, it is important to acknowledge that explainable AI still lacks standardized methodologies and consensus on how to assess the real-world utility of these tools—representing both a limitation of this work and a broader challenge for the community.

## 5. Conclusions

This study presents a comprehensive analyte importance analysis for machine learning-based detection of wrong blood in tube (WBIT) errors using complete blood count (CBC) data. By performing exhaustive feature selection for all analyte combinations and evaluating multiple importance metrics—SHAP, permutation importance, and mean decrease in impurity—we were able to identify the analytes with the highest predictive utility. MCV and RDW consistently ranked as the most impactful features, providing robust classification performance even when used in smaller subsets.

Our approach extends previous studies by incorporating model interpretability at both global and local levels. We increased transparency through visual explanations in two and three dimensions, illustrating how the models distinguish WBIT from non-WBIT cases based on feature space distribution. These visualization techniques not only demystify the decision boundaries learned by the classifiers, but also provide insight into misclassifications and the trade-off between precision and recall.

In this way, we provide a methodological framework for feature selection in ML-based WBIT detection systems, enabling better transferability of the model to other laboratories with different test profiles. The results show that high accuracy can be achieved with a minimal subset of routinely available analytes, making the method potentially suitable for integration into clinical practice.

Furthermore, this work provides a structured overview of the current state of research in ML-based WBIT detection, identifying gaps in simulation methods, algorithm selection, and confirmation strategies for use in practice. Future work should focus on utilizing advanced deep learning architectures for tabular data, overcoming challenges in clinical verification, and integrating adaptive techniques for missing data and patient-specific time series patterns. Ultimately, improving WBIT detection can increase patient safety by reducing pre-analytical errors in laboratory diagnostics.

## Figures and Tables

**Table 1:** Baseline characteristics of the filtered dataset.

Analyte	Mean	Std Dev	Median	IQR	Min	Max
ERY	4.11	0.75	4.1	1	0.7	10
HK	37.02	6.52	37.3	9	5.7	84.5
LEUKO	9.03	6.27	8	4.5	0.1	571.7
HB	12.27	2.24	12.4	3.2	2.1	28.1
PLT	255.38	112.85	238	117	3	2689
MCV	90.7	7.42	90.5	8.2	37.5	144.7
MCHC	33.13	1.27	33.2	1.5	23.1	152.1
MCH	30.05	2.65	30.2	2.8	12	104.8
RDW	14.89	2.01	14.4	2.2	10	40.3
age	63.78	20.18	68	26	0	106

ERY: red blood cells ($\times 10^{6}/\mu L$), HB: hemoglobin (g/dL), HK: hematocrit (%), MCH: mean corpuscular hemoglobin (pg), MCV: mean corpuscular volume (fL), MCHC: mean corpuscular hemoglobin concentration (g/dL), RDW: red blood cell distribution width (%), PLT: platelets ($\times 10^{3}/\mu L$), LEUKO: white blood cells ($\times 10^{3}/\mu L$), IQR: interquartile range.

**Table 2:** Baseline counts of the *filtered* dataset.

Category	Number of Samples
All samples	523,844 (100.00%)
Male:	260,041 (49.64%)
Female:	263,690 (50.34%)
Unidentified:	113 (0.02%)
Time difference from the last sample	
<1 day:	179,139 (34.20%)
<1 week:	112,293 (21.44%)
<1 month:	65,081 (12.42%)
>1 month:	167,331 (31.94%)
Number of patients w.r.t. number of their samples	
2 samples:	22,946 (29.66%)
3–5 samples:	27,992 (36.19%)
>5 samples:	26,417 (34.15%)

**Table 3:** Exhaustive feature selection. For each analyte set size *n*, models are trained and evaluated using every possible combination of analytes. Scores for the top 5 combinations for each *n*, sorted with respect to accuracy, are shown.

n	Analyte Combination	Accuracy	f1	roc auc	ppv	Sensitivity
1	MCV	83.12 ± 0.12	81.08 ± 0.16	88.69 ± 0.14	89.86 ± 0.57	74.5 ± 0.3
	MCH	82.38 ± 0.18	79.83 ± 0.32	87.72 ± 0.19	88.93 ± 0.23	71.82 ± 0.81
	RDW	80.62 ± 0.18	78.08 ± 0.23	86.44 ± 0.13	86.55 ± 0.17	71.11 ± 0.32
	HB	75.9 ± 0.25	72.56 ± 0.4	81.58 ± 0.33	81.96 ± 0.11	66.75 ± 0.56
	ERY	75.62 ± 0.18	71.75 ± 0.27	81.09 ± 0.21	81.08 ± 0.06	65.67 ± 0.65
2	MCV, RDW	88.74 ± 0.1	88.01 ± 0.14	94.43 ± 0.14	91.28 ± 0.12	85.09 ± 0.16
	MCH, RDW	88.43 ± 0.1	87.64 ± 0.12	94.14 ± 0.05	91.19 ± 0.07	84.3 ± 0.27
	ERY, MCH	87.64 ± 0.17	86.63 ± 0.25	93.35 ± 0.16	91.13 ± 0.15	82.59 ± 0.37
	HB, MCH	87.48 ± 0.14	86.44 ± 0.22	93.26 ± 0.16	91.04 ± 0.22	82.47 ± 0.51
	ERY, MCV	87.4 ± 0.17	86.4 ± 0.22	93.12 ± 0.16	90.95 ± 0.19	82.41 ± 0.49
3	HB, MCV, RDW	90.12 ± 0.21	89.8 ± 0.17	95.68 ± 0.16	91.42 ± 0.17	89.48 ± 0.18
	ERY, MCV, RDW	90.06 ± 0.14	89.7 ± 0.27	95.59 ± 0.16	91.08 ± 0.18	89.39 ± 0.32
	HK, MCV, RDW	90.04 ± 0.3	89.63 ± 0.16	95.52 ± 0.05	90.83 ± 0.1	89.03 ± 0.17
	ERY, MCH, RDW	89.75 ± 0.08	89.22 ± 0.08	95.24 ± 0.16	90.63 ± 0.21	88.83 ± 0.34
	HB, MCH, RDW	89.61 ± 0.15	89.18 ± 0.13	95.2 ± 0.14	90.46 ± 0.12	88.26 ± 0.26
4	HB, PLT, MCV, RDW	92.35 ± 0.2	92.08 ± 0.21	97.24 ± 0.11	92.71 ± 0.16	91.68 ± 0.29
	HK, PLT, MCV, RDW	92.31 ± 0.18	92.04 ± 0.19	97.21 ± 0.11	92.67 ± 0.15	91.67 ± 0.29
	ERY, PLT, MCV, RDW	92.17 ± 0.16	91.89 ± 0.2	97.15 ± 0.12	92.56 ± 0.16	91.61 ± 0.28
	HK, MCV, MCH, RDW	92.12 ± 0.14	91.79 ± 0.15	96.98 ± 0.08	92.51 ± 0.11	91.1 ± 0.3
	HK, MCV, MCHC, RDW	92.05 ± 0.16	91.76 ± 0.16	96.91 ± 0.11	92.49 ± 0.17	91.03 ± 0.21
5	HB, PLT, MCV, MCH, RDW	93.46 ± 0.13	93.25 ± 0.16	97.86 ± 0.08	93.59 ± 0.17	92.91 ± 0.25
	HK, PLT, MCV, MCH, RDW	93.46 ± 0.18	93.24 ± 0.22	97.84 ± 0.09	93.59 ± 0.2	92.86 ± 0.25
	HK, PLT, MCV, MCHC, RDW	93.41 ± 0.2	93.18 ± 0.19	97.83 ± 0.08	93.55 ± 0.18	92.84 ± 0.3
	HB, PLT, MCV, MCHC, RDW	93.4 ± 0.17	93.15 ± 0.14	97.83 ± 0.09	93.54 ± 0.19	92.84 ± 0.3
	ERY, PLT, MCV, MCH, RDW	93.37 ± 0.12	93.15 ± 0.2	97.82 ± 0.09	93.53 ± 0.17	92.8 ± 0.25
6	LEUKO, HB, PLT, MCV, MCH, RDW	93.75 ± 0.14	93.55 ± 0.15	98.07 ± 0.08	93.83 ± 0.14	93.22 ± 0.18
	ERY, LEUKO, PLT, MCV, MCH, RDW	93.68 ± 0.15	93.49 ± 0.17	98.06 ± 0.09	93.82 ± 0.13	93.21 ± 0.26
	HK, LEUKO, PLT, MCV, MCH, RDW	93.68 ± 0.17	93.48 ± 0.16	98.05 ± 0.08	93.81 ± 0.13	93.2 ± 0.26
	LEUKO, HB, PLT, MCV, MCHC, RDW	93.67 ± 0.14	93.45 ± 0.15	98.03 ± 0.08	93.75 ± 0.13	93.17 ± 0.26
	HK, LEUKO, PLT, MCV, MCHC, RDW	93.64 ± 0.13	93.4 ± 0.18	98.01 ± 0.08	93.71 ± 0.14	93.17 ± 0.22
7	ERY, LEUKO, HB, PLT, MCV, MCH, RDW	93.79 ± 0.14	93.57 ± 0.14	98.11 ± 0.09	93.91 ± 0.15	93.27 ± 0.17
	ERY, HK, LEUKO, PLT, MCV, MCH, RDW	93.76 ± 0.15	93.56 ± 0.14	98.09 ± 0.09	93.89 ± 0.17	93.23 ± 0.23
	LEUKO, HB, PLT, MCV, MCHC, MCH, RDW	93.74 ± 0.15	93.55 ± 0.18	98.08 ± 0.08	93.89 ± 0.1	93.22 ± 0.23
	HK, LEUKO, HB, PLT, MCV, MCH, RDW	93.73 ± 0.17	93.52 ± 0.17	98.08 ± 0.09	93.86 ± 0.19	93.21 ± 0.26
	HK, LEUKO, PLT, MCV, MCHC, MCH, RDW	93.72 ± 0.14	93.51 ± 0.16	98.07 ± 0.09	93.83 ± 0.16	93.2 ± 0.27
8	ERY, HK, LEUKO, HB, PLT, MCV, MCH, RDW	93.79 ± 0.16	93.58 ± 0.17	98.11 ± 0.08	93.94 ± 0.12	93.27 ± 0.18
	ERY, LEUKO, HB, PLT, MCV, MCHC, MCH, RDW	93.77 ± 0.15	93.56 ± 0.14	98.11 ± 0.1	93.93 ± 0.13	93.2 ± 0.21
	HK, LEUKO, HB, PLT, MCV, MCHC, MCH, RDW	93.74 ± 0.16	93.54 ± 0.16	98.09 ± 0.09	93.91 ± 0.14	93.19 ± 0.23
	ERY, HK, LEUKO, PLT, MCV, MCHC, MCH, RDW	93.72 ± 0.15	93.52 ± 0.17	98.09 ± 0.09	93.89 ± 0.14	93.18 ± 0.23
	ERY, HK, LEUKO, HB, PLT, MCV, MCHC, RDW	93.68 ± 0.19	93.51 ± 0.16	98.08 ± 0.08	93.81 ± 0.11	93.17 ± 0.24
9	ERY, HK, LEUKO, HB, PLT, MCV, MCHC, MCH, RDW	93.75 ± 0.15	93.54 ± 0.16	98.09 ± 0.09	93.82 ± 0.1	93.23 ± 0.21

**Table 4:** The models are evaluated that are trained on analyte combinations that yield the best performance for each *n*. Positive predictive value (PPV) scores are abbreviated such that PPV@S0.8E0.005 corresponds to PPV at 0.8 sensitivity and an assumed WBIT error rate of 0.005. ROC AUC and F1 are given with 95% bootstrap confidence intervals over 1000 iterations.

*n*	PPV@S0.8E0.005	PPV@S0.5E0.005	ROC AUC	F1
9	17.25 (14.01, 26.0)	34.17 (32.32, 45.0)	97.92 (97.83, 98.0)	92.8 (92.61, 92.97)
8	19.09 (14.79, 25.93)	42.29 (32.35, 46.07)	97.93 (97.84, 98.01)	92.82 (92.63, 93.0)
7	15.78 (13.93, 26.28)	35.24 (32.37, 44.49)	97.92 (97.83, 97.99)	92.86 (92.68, 93.04)
6	19.29 (14.04, 25.49)	32.82 (29.88, 43.28)	97.89 (97.8, 97.97)	92.76 (92.58, 92.93)
5	16.74 (12.8, 22.86)	35.47 (28.5, 39.87)	97.63 (97.54, 97.72)	92.46 (92.26, 92.64)
4	15.65 (11.02, 20.16)	25.47 (24.68, 35.55)	97.26 (97.15, 97.36)	91.93 (91.73, 92.13)
3	8.75 (7.0, 16.64)	23.45 (22.28, 33.33)	95.63 (95.49, 95.77)	89.56 (89.34, 89.79)
2	7.66 (5.12, 11.66)	17.7 (15.99, 20.09)	94.42 (94.26, 94.58)	88.03 (87.79, 88.26)
1	4.9 (1.62, 5.07)	29.58 (19.18, 37.8)	89.28 (89.06, 89.52)	81.65 (81.36, 81.97)

**Table 5:** Analyte importance analysis. For each analyte set size *n*, the models are analyzed that are trained on analyte combinations that yield the best performance according to exhaustive search (see Table 3). Three feature importance metrics are measured (see Section 3) and sorted with respect to MDI. Ranges: MDI, SHAP: ≥0; PI: 0–1. Higher values indicate greater importance.

Set Size (*n*)	Analyte	MDI	SHAP	PI
9	MCV	0.23	0.15	0.06
	MCH	0.21	0.14	0.03
	RDW	0.19	0.13	0.1
	HB	0.1	0.06	0.02
	PLT	0.08	0.06	0.03
	ERY	0.06	0.04	0
	HK	0.06	0.03	0
	LEUKO	0.04	0.02	0
	MCHC	0.03	0.01	0
8	MCV	0.24	0.15	0.08
	RDW	0.19	0.13	0.1
	MCH	0.18	0.12	0.03
	HB	0.11	0.07	0.02
	PLT	0.09	0.06	0.03
	ERY	0.07	0.05	0
	HK	0.08	0.05	0
	LEUKO	0.04	0.02	0
7	MCV	0.25	0.16	0.1
	MCH	0.21	0.14	0.04
	RDW	0.2	0.13	0.1
	HB	0.12	0.08	0.03
	ERY	0.08	0.06	0.01
	PLT	0.09	0.06	0.03
	LEUKO	0.04	0.02	0
6	MCV	0.27	0.17	0.1
	MCH	0.21	0.14	0.05
	RDW	0.21	0.14	0.1
	HB	0.15	0.1	0.09
	PLT	0.11	0.07	0.04
	LEUKO	0.05	0.03	0
5	MCV	0.28	0.17	0.1
	MCH	0.25	0.16	0.05
	RDW	0.22	0.14	0.11
	HB	0.15	0.1	0.09
	PLT	0.1	0.06	0.04
4	MCV	0.4	0.23	0.17
	RDW	0.29	0.18	0.12
	HB	0.18	0.11	0.09
	PLT	0.13	0.07	0.05
3	MCV	0.44	0.23	0.18
	RDW	0.3	0.18	0.14
	HB	0.25	0.15	0.1
2	MCV	0.54	0.25	0.21
	RDW	0.46	0.22	0.17

## Data Availability

The original data presented in the study are openly available in Zenodo at https://doi.org/10.5281/zenodo.15674541 accessed on 25 June 2025. To mitigate re-identification risks associated with rare analyte patterns, the published dataset includes only samples retained after the filtering steps described in Section 2.3.

## Abbreviations

The following abbreviations are used in this manuscript:

AI	Artificial Intelligence
AUC	Area Under the Receiver Operating Characteristic Curve
CBC	Complete Blood Count
CI	Confidence Interval
EDTA	Ethylenediaminetetraacetic Acid
ERY	Erythrocytes (Red Blood Cells)
HB	Hemoglobin
HK	Hematocrit
IQR	Interquartile Range
LEUKO	Leukocytes (White Blood Cells)
LIS	Laboratory Information System
LOF	Local Outlier Factor
LSTM	Long Short-Term Memory
MCH	Mean Corpuscular Hemoglobin
MCHC	Mean Corpuscular Hemoglobin Concentration
MCV	Mean Corpuscular Volume
MDI	Mean Decrease in Impurity
ML	Machine Learning
PI	Permutation Importance
PLT	Platelets
PPV	Positive Predictive Value
RFC	Random Forest Classifier
ROC	Receiver Operating Characteristic
SHAP	SHapley Additive exPlanations
SVM	Support Vector Machine
WBIT	Wrong Blood in Tube
